# Targeting Crosstalk of Signaling Pathways among Muscles-Bone-Adipose Tissue: A Promising Therapeutic Approach for Sarcopenia

**DOI:** 10.14336/AD.2023.00903

**Published:** 2024-08-01

**Authors:** Ashish Ranjan Sharma, Srijan Chatterjee, Yeon-Hee Lee, Sang-Soo Lee

**Affiliations:** Institute for Skeletal Aging & Orthopedic Surgery, Hallym University-Chuncheon Sacred Heart Hospital, Chuncheon-si, 24252, Gangwon-do, Republic of Korea.

**Keywords:** Sarcopenia, muscle, bone, adipose tissue, crosstalk, therapeutics

## Abstract

The aging process is associated with the development of a wide range of degenerative disorders in mammals. These diseases are characterized by a progressive decline in function at multiple levels, including the molecular, cellular, tissue, and organismal. Furthermore, it is responsible for various healthcare costs in developing and developed countries. Sarcopenia is the deterioration in the quality and functionality of muscles, which is extremely concerning as it manages many functions in the human body. This article reviews the molecular crosstalk involved in sarcopenia and the specific roles of many mediator molecules in establishing cross-talk between muscles, bone, and fatty tissues, eventually leading to sarcopenia. Besides, the involvement of various etiological factors, such as neurology, endocrinology, lifestyle, etc., makes it exceedingly difficult for clinicians to develop a coherent hypothesis that may lead to the well-organized management system required to battle this debilitating disease. The several hallmarks contributing to the progression of the disease is a vital question that needs to be addressed to ensure an efficient treatment for sarcopenia patients. Also, the intricate molecular mechanism involved in developing this disease requires more studies. The direct relationship of cellular senescence with aging is one of the pivotal issues contributing to disease pathophysiology. Some patented treatment strategies have been discussed, including drugs undergoing clinical trials and emerging options like miRNA and protein-enclosed extracellular vesicles. A clear understanding of the secretome, including the signaling pathways involved between muscles, bone, and fatty tissues, is extremely beneficial for developing novel therapeutics for curing sarcopenia.

## 1. Introduction

Sarcopenia is a pathological condition mostly prevalent in older ages and is characterized by a significant loss in the mass and function of the skeletal muscle cells [1]. Muscle mass contributes to approximately 60% of the body weight. Thus, the alteration in the functionality of this tissue might be extremely detrimental for people, especially aging adults. The loss of functionality of the muscle tissues can lead to severe health problems, including frailty and disability [2-4]. Besides, the victims of sarcopenia also suffer an extremely poor life characterized by increased risks of several metabolic diseases and fractures in the bone, even leading to mortality [5].

In 2000, WHO denoted sarcopenia as one of the potent factors contributing to several morbidities in aging adults. However, leading a healthy lifestyle can sometimes be beneficial to escape from these age-related ailments. Sarcopenia is denoted as M62.84 according to the ICD-10-CM code [6]. As per WHO, the ICD-10 is abbreviated as the “International Classification of Diseases External 10th Revision” (ICD-10). It was executed in 1999 to assess the mortality coding and the classification from the death certificates in the U.S. [7]. A significant turning point in the path towards identifying sarcopenia as a disease was the adoption of the ICD-10-CM classification. This was analogous to the much earlier recognition of osteoporosis as a disease state [8]. As a result, medical professionals and pharmaceutical companies became more interested in diagnosing and creating therapeutics to treat sarcopenia [9, 10]. The increased pressure of globalization has drifted the focus of individuals more on healthspan than lifespan, highlighting the need to work for society [11].

Generally, the case studies indicate that approximately 5% to 13% of patients over the age of 60 and 11% to 50% of patients over 80 are victims of sarcopenia [12]. The disease is found to affect both males and females in a similar ratio [13]. The chances of developing sarcopenia are higher for those patients suffering from chronic ailments, namely Chronic Kidney Disease (CKD), Human Immunodeficiency Virus (HIV), cancer, Chronic Obstructive Pulmonary Disease (COPD), etc. Several aspects of developing sarcopenia are still to be understood. Factors, including ethnicity, method of diagnosis, and the subjects chosen for the study, make it very tough to provide a clear hypothesis regarding the prevalence of sarcopenia, making it a concerning health issue in the public sector [14]. According to several reports based on population studies made by WHO, sarcopenia is found in a large percentage of the population, approximately 50 million, and the surge will be up to 200 million in the next four decades [15].

**Figure 1.**
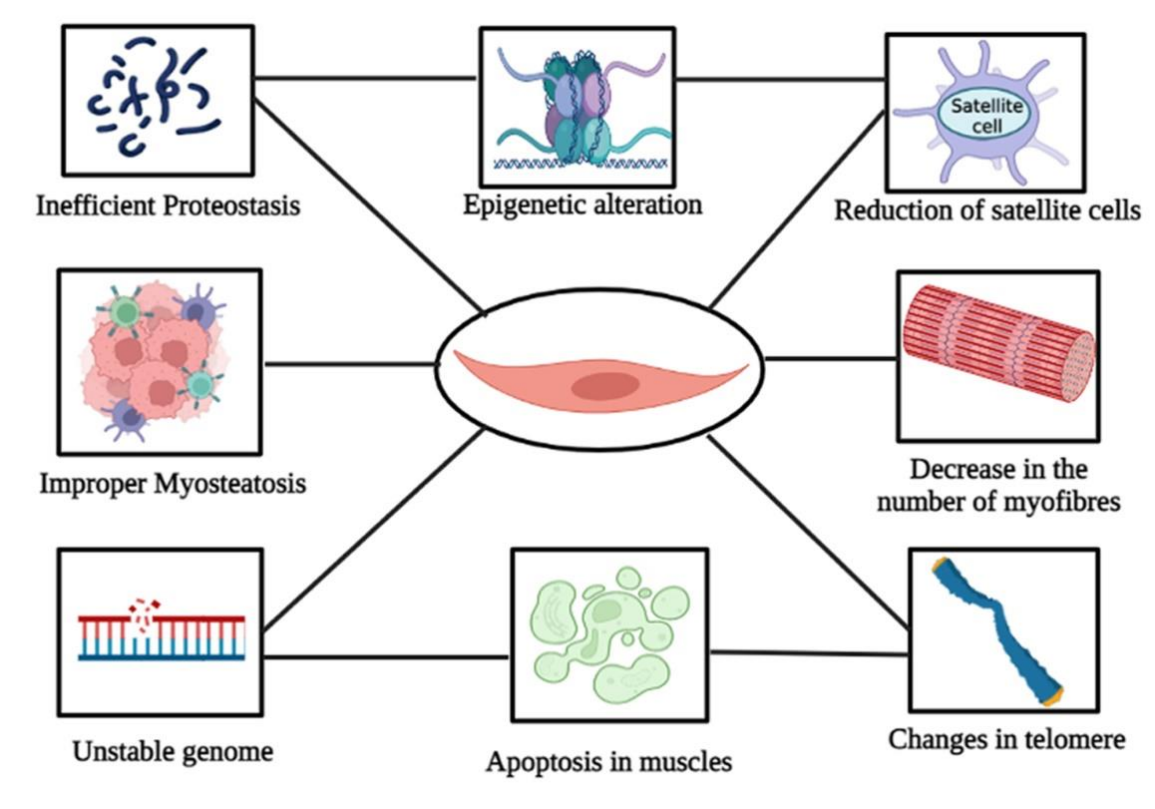
Some of the significant hallmarks of sarcopenia caused by the rapid damage in the cells leading to changes in telomere, improper myosteatosis, and proteostasis, reduction in satellite cells and myofibres, muscle apoptosis, and epigenetic alteration.

The progression of sarcopenia is influenced by various etiological variables, including endocrine changes, neurological causes, and dietary and lifestyle modifications associated with sedentary habits. A deviation from a balanced diet or overnutrition might cause sarcopenia [16, 17]. Some hypothesized mechanisms responsible for sarcopenia include apoptosis, recruitment of new muscle cells, remodeling of the muscle tissues, protein turnover, and degeneration of several alpha-motor neurons [18]. As cellular and molecular pathways are still not thoroughly understood, there is no proven cure for this disease. Thus, knowing the precise molecular mechanism involved in sarcopenia is essential [19]. In this review, we have tried to recapitulate the molecular mechanism and the signaling pathways, including the mediator molecules involved in crosstalk among cells like skeletal, bone, and adipose cells, contributing to sarcopenia. The intricated relationship between the bones and muscles makes it difficult for scientists to invent new therapeutics against this musculoskeletal disorder. Nevertheless, a broader prospect of research that points out novel biomarkers against sarcopenia will surely be beneficial in treating this disease in the near future. This review will also offer some hallmarks of sarcopenia, which can be an effective method for medical practitioners to get a clear idea of whether the patient is sarcopenic. A proper diagnosis with an effective treatment method will help society eliminate this detrimental disorder.

## 2. Molecular mechanism involved in sarcopenia

Aging is considered a primary hallmark of sarcopenia, which is defined by rapid damage in the cells leading to an unstable genome, changes in the telomere, inefficient proteostasis, and epigenetic alterations (Fig. 1). The molecular or cellular signaling in sarcopenia is mainly characterized by external environmental changes and internal skeletal muscle alterations [20]. The two molecules that are engaged in the molecular signaling of sarcopenia include Smad and Akt [21, 22]. The involvement of several hormones in muscle wasting cannot be overlooked. For instance, glucocorticoids mediate the atrophy of muscles as the person ages [23-25]. These hormones involve anabolic pathways like IGF-1 or insulin [23, 26-28]. One of the most important mechanisms behind aging involves the interference of several free radicals. This interference leads to the massive accumulation of oxidative stress in the muscle cells, leading to the malfunctioning of the muscles. Due to the aging of muscle cells, cells fail to recognize these accumulated signaling molecules, giving rise to the loss of functionality [29]. Sarcopenia is further exacerbated by a decrease in the number of satellite cells [30]. The elevated Smad4 in aging adults hinders the satellite cells from differentiating during the process of muscle regeneration, making them extremely inefficient in regenerating and self-renewing [31]. Removing some redox-dependent transcription factors like Nrf2 triggers apoptosis and decreases the population of stem cells, weakening the muscles' potential to regenerate during oxidative stress [32].

Loss of mass and function of skeletal muscles results in the successive failure of repairing the damaged cells. The huge production of cytokines may be responsible for this phenomenon. Some inflammatory cytokines interfere with the IGF-I signaling pathway of the skeletal muscle cells and develop sarcopenia [33]. Increased levels of myogenin, an essential factor for myoblast differentiation, are responsible for the loss of muscle mass in many circumstances like starvation, atrophy, denervation, etc. [34]. Evidence suggests that a greater prevalence of misfolded protein, followed by the altered functional landscape of mitochondria in sarcopenic patients, enhances the possibility of a high amount of reactive oxygen species (ROS) accumulation, negatively impacting the functions of the muscles [35]. Moreover, mitochondria are also responsible for the loss of fiber in sarcopenic patients, as they can activate the apoptotic signaling cascade. The impaired function of mitochondria can be understood when it starts releasing cytochrome C [36]. The cytochrome C released from mitochondria with some ancillary factors promotes apoptosis of the fiber cells in sarcopenic patients [37]. The mTOR is a kinase molecule that regulates hypertrophy of the skeletal muscles [38-40]. Likewise, the mTOR is required for efficient modulation in protein synthesis. Several hormones like insulin, IGF-I, and testosterone initiate the mTOR signaling by implementing protein kinase B [41]. Protein kinase B also mediates the proliferation of the muscle cells by the action of BCAAs and IGF-I [41, 42].

**Figure 2.**
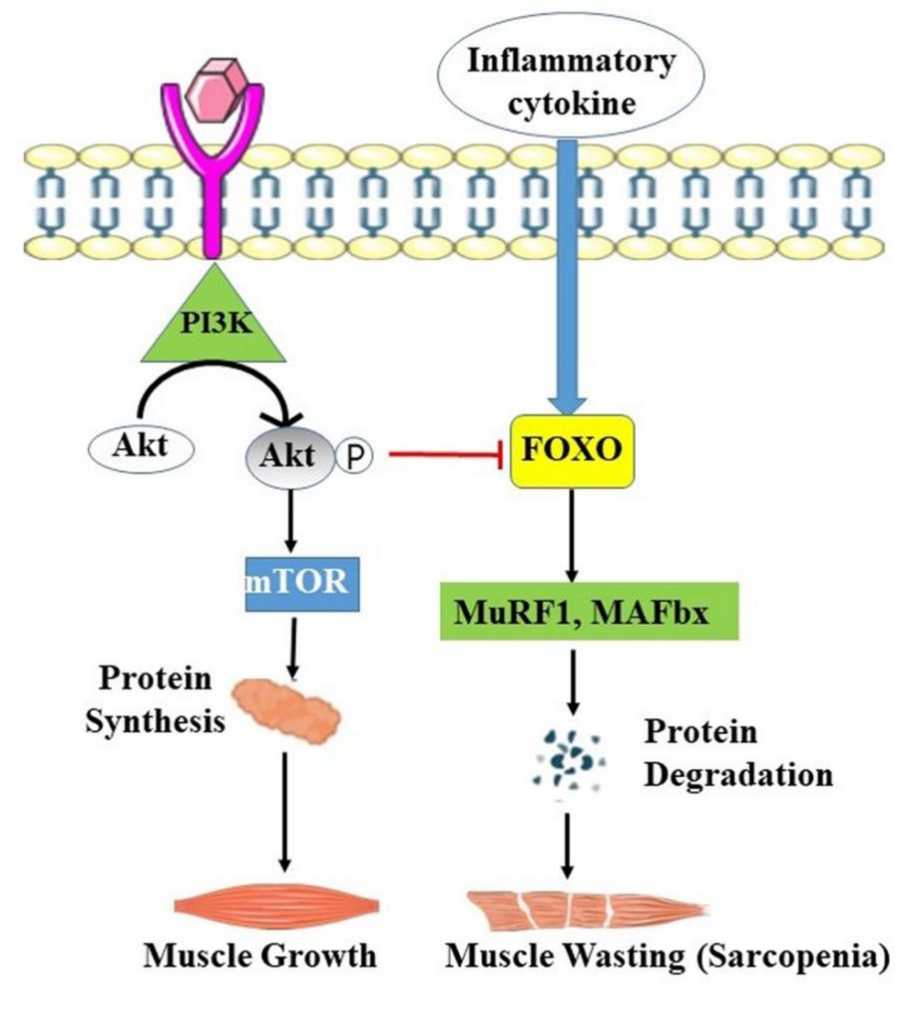
**The molecular mechanism involved in normal muscle growth and muscle wasting**. The Akt pathway is mainly responsible for synthesizing various muscle proteins by activating mTOR, which also downregulates the FOXO transcription, leading to the recruitment of MuRF1 and MAFbx and, thus, sarcopenia.

Most importantly, the size of the skeletal muscles is regulated by the Akt signaling pathway under the influence of insulin and IGF-1. These molecules promote the synthesis of proteins along with muscle hypertrophy. They react with the tyrosine kinase receptors, leading to the phosphorylation of IRS-1 (Insulin-like growth factor 1) [43]. Scientists also highlighted that the Akt level was extremely low in the skeletal muscles of old-aged adults [44, 45]. The Akt pathway mainly synthesizes various muscle proteins [46]. The loss of muscle mass is well understood by observing the levels of the positive and negative mediator molecules. For instance, the downregulated level of irisin and follistatin or the upregulation of activin A, TGF-β, and myostatin indicates a decrease in muscle mass and thus can serve as the biomarker for diagnosing sarcopenia [47-49]. Insulin or IGF-1 helps in regulating muscle mass by activating the Akt molecule. The Akt molecule, in turn, activates mTORC1 through the IRS1-PI3K axis. The mTORC1 triggers the phosphorylation of 4E-binding protein (4E-BP) and ribosomal protein S6 kinase 1 (S6K). Increased synthesis of the muscle protein results when GSK3 no longer inhibits eIF2B, regarded as an initiation factor for the translation process. Activation of the Akt signaling pathway downregulates the transcription rate of the *FOXO* gene, resulting in a decrease in the production of MuRF1 and MAFbx. However, the involvement of Smad proteins in the Akt signaling pathway reverses the effect of Akt on forkhead box protein O (FOXO), altering the production rate of MuRF1 and MAFbx [50] (Fig. 2).

A wide family of proteins named the FOXO comprises FOXO transcription factors [51] and is protected by a DNA-binding domain. FOXO1, FOXO3, and FOXO4 are members of the FOXO family that maintain the physiology of skeletal muscles [52]. A majority of FOXOs are present in the nucleus, where they control the production of several downstream signaling proteins. However, FoxOs are redirected into the cytosol when they are phosphorylated, primarily by the Akt, and incapable of transcribing the genes responsible for causing muscle atrophy. Recent research has shown that FoxO1 impairs the signaling pathway by decreasing the levels of regulatory-associated protein of mammalian target of rapamycin (RAPTOR) and mTOR, as well as increasing the rate of expression followed by the decrease in the phosphorylation of the 4E-BP1 protein, a translational repressor [51].

## 3. Cellular senescence and sarcopenia

**Figure 3.**
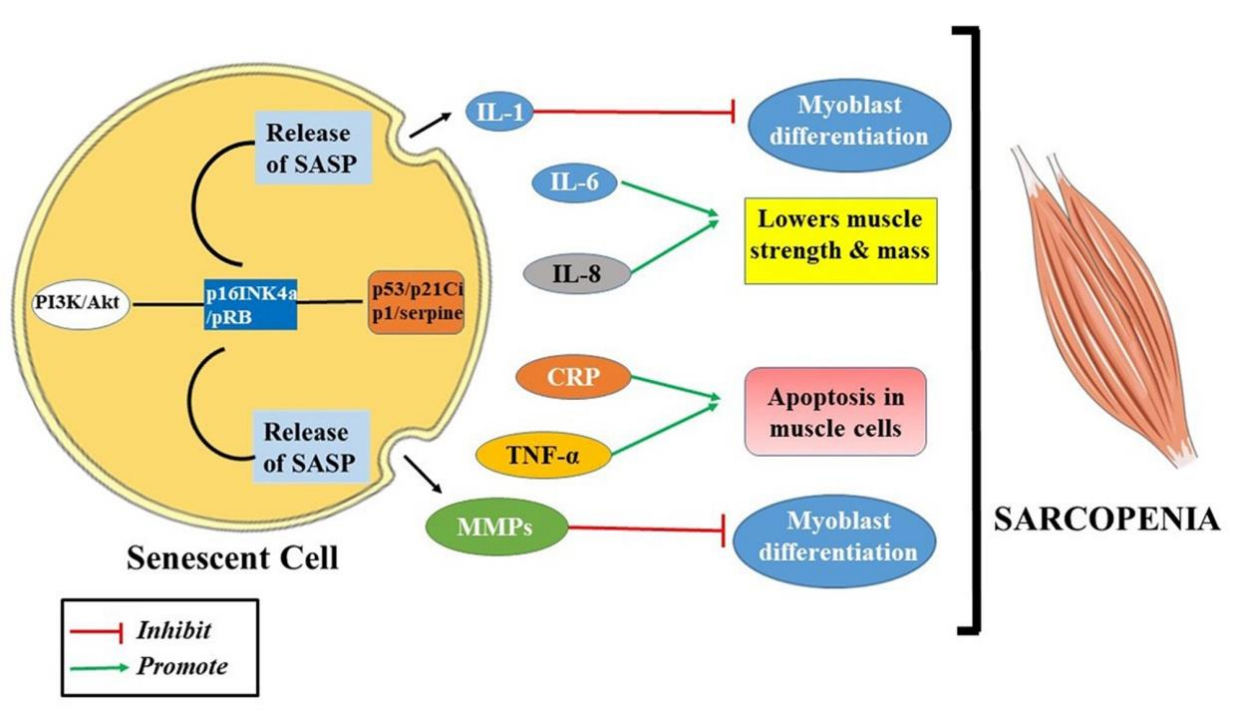
**The two ways by which cellular senescence leads to sarcopenia**. It upregulates the anti-apoptotic pathways like PI3K/Akt, p16INK4a/PRB, p53/p21Cip1/serpine or increases the number of senescence-associated secretory phenotype (SASP) like IL-1, MMPs, CRP, IL-6, IL-8 which affects the normal functioning of muscle and leads to the development of sarcopenia.

An abundance of senescent cells has been shown to have a causative effect on the acceleration of tissue aging, potentially leading to chronic illnesses and cellular senescence [53]. By preventing the differentiation of cancer cells, cellular senescence inhibits tumorigenesis. On the contrary, senescent cells damage tissue integrity and promote the progression of numerous age-related diseases, such as sarcopenia [54, 55]. By 2021, sarcopenia has been regarded as a geriatric disease that affects the musculoskeletal system and possesses multiple causes, including systemic inflammation, loss of neuromuscular functions, decreased levels of anabolic hormones, malfunctioning of the mitochondria, and oxidative stress [20]. However, the exact factors or pathways responsible for developing sarcopenia are yet to be deciphered. Senescent cells are involved in the upregulation of several anti-apoptotic pathways, including the PI3K/Akt, p53/p21Cip1/serpine, and p16INK4a/pRB pathways [56, 57] (Fig. 3).

These pathways interact with one another and eventually create dependent networks. Numerous studies have identified various phenotypes that manifest during cellular senescence. These include DNA damage, also known as DNA damage response (DDR), phenotypes induced by telomere erosion [58], reactive metabolites, proteotoxic stress, oncogenic mutations, epigenetic stress, mitogens/IGF-1, cyclin-dependent kinase inhibitors (CDKi) damage-associated molecular patterns (DAMPs), and other factors [59]. To date, it is recommended that interventions on the signaling pathways be made in light of the cellular senescence mechanisms in sarcopenia that can reveal the therapeutic prospect of treating this multifactorial disease. To be more precise, it can be asserted that there exists a direct association between cellular senescence and sarcopenia, hence triggering the pathological manifestations of this particular ailment. [60]. The multifaceted nature of senescence has been growing quickly recently, but more study is still required to fully comprehend the myriad biological functions of senescent cells and how they affect the pathophysiology of sarcopenia [57]. Studies have demonstrated that the use of pharmacological inhibitors SB202190 (EMD Chemicals) or SB203580 (Tocris) can effectively mitigate age-associated sarcopenia and offer a promising therapeutic approach for addressing progressive muscle wasting [61, 62]. The study conducted by McHugh et al. involved a comprehensive examination of the impacts associated with administering a particular inhibitor peptide that specifically targets FOXO4 in mice [63]. They found that this treatment can effectively delay various aging phenotypes. One notable effect observed was a significant decrease in the expression of p21CIP1 in senescent cells. This decrease is attributed to the role of FOXO4 in preventing the localization of p53 to the nucleus, thereby protecting against the activation of the p53-mitochondrial signaling axis and subsequent apoptosis [63, 64]. According to Zhu et al., in 4 months old mice, localized DNA damage was induced by irradiating one hindlimb. The co-administration of dasatinib and quercetin in a singular dosage reduced the expression of the senescence marker p16 in the quadriceps muscle. Additionally, this treatment led to an improvement in treadmill performance, which was observed at both 5 days and seven months after the treatment when compared to the control group that received a vehicle [65]. The study involved the observation of 20-month-old mice that underwent an 8-week treatment with a newly developed prodrug called senescence-specific killing compound 1 (SSK1), which is responsive to SA-βgal. The results indicated that the treated mice exhibited enhanced exercise capacity, coordination, and balance, along with increased muscle strength, compared to those who received a vehicle treatment. The observed enhancement can be ascribed to the targeted nature of SSK1, which exploits the increased activity of the lysosomal enzyme in senescent cells, thus augmenting the specificity of the medication. [66]. There is a prevailing expectation that anabolic hormones, including growth hormones, testosterone, and selective androgen receptor modulators (SARMs), can potentially enhance lean body mass (LBM). However, obtaining more evidence of a more robust nature is imperative to establish a greater level of assurance regarding their efficacy in reversing sarcopenia [67, 68]. In a single high-quality randomized controlled trial (RCT) including sarcopenic older female participants, the administration of MK-0773, a selective androgen receptor modulator (SARM), for a duration exceeding six months, resulted in an observed augmentation of skeletal muscle mass.

According to preliminary research, sarcopenia's recognizable muscle-fiber thinning is triggered by accelerated senescence of both the muscle cells and their Senescence-associated Secretory Phenotype (SASP). A recent study investigated whether the "bystander effect" and senescent-like signaling would cause SnC-implanted close to skeletal muscle to damage the healthy surrounding muscle cells [69]. Elevated senescent markers and weakening muscle fibers are signs of sarcopenia, predominantly caused by SnC implantation next to skeletal muscle. The upregulation of SASP factors has also been associated with sarcolipin and other factors identified as promoters of skeletal muscle fibrosis, ultimately contributing to the development of sarcopenia. [70]. In addition to age-related muscle loss, there is an ongoing exploration of the potential applications of senotherapeutics in the field of muscle illnesses. The presence of p16 has been observed in skeletal biopsy specimens of children and young adults (aged 3-33 years) with Duchenne muscular dystrophy (DMD). However, it is not detected in individuals of the same age group who do not have the condition [71].

Cellular senescence within the aging skeletal muscle milieu can provide insight into sarcopenia's underlying causes. Nevertheless, potential problems may arise from interventions addressing cellular senescence and the SASP in sarcopenia. When examining therapeutic approaches for sarcopenia, it is important to consider the potential effects of suppressing regulators of SASP, such as the inhibition of p38MAPK. These inhibitions may inadvertently suppress the p53 and p16 pathways, thereby impeding the advantageous impacts of cellular senescence on various diseases, such as cancer suppression. Similarly, the mediators that regulate the SASP are implicated in numerous cellular processes. Hence, interventions targeting the modulation of the SASP regulatory network have the potential to influence various other biological systems. Further research is necessary to elucidate the specific processes or synergistic interactions contributing to the SASP. Furthermore, it is important to address several crucial factors concerning the susceptibility of different populations of skeletal muscle cells to senescence. These factors include evaluating the candidate compound's bioavailability specifically to skeletal muscle, determining the potential impact of senescence and the secretion of SASP in specific cellular subpopulations on age- and disease-related changes within the tissue, and exploring how the selected drug's biological effects can be observed through a decrease in the prevalence of senescent cells within a cell population or, in cases of high abundance, at the tissue level [72]. Therefore, in addition to focusing on the aging process of muscle tissues, it is imperative to develop a comprehensive understanding of the molecular and cellular communication involved in the underlying pathophysiology of sarcopenia.

## 4. Molecules involved in bone-muscle crosstalk in sarcopenia

**Figure 4.**
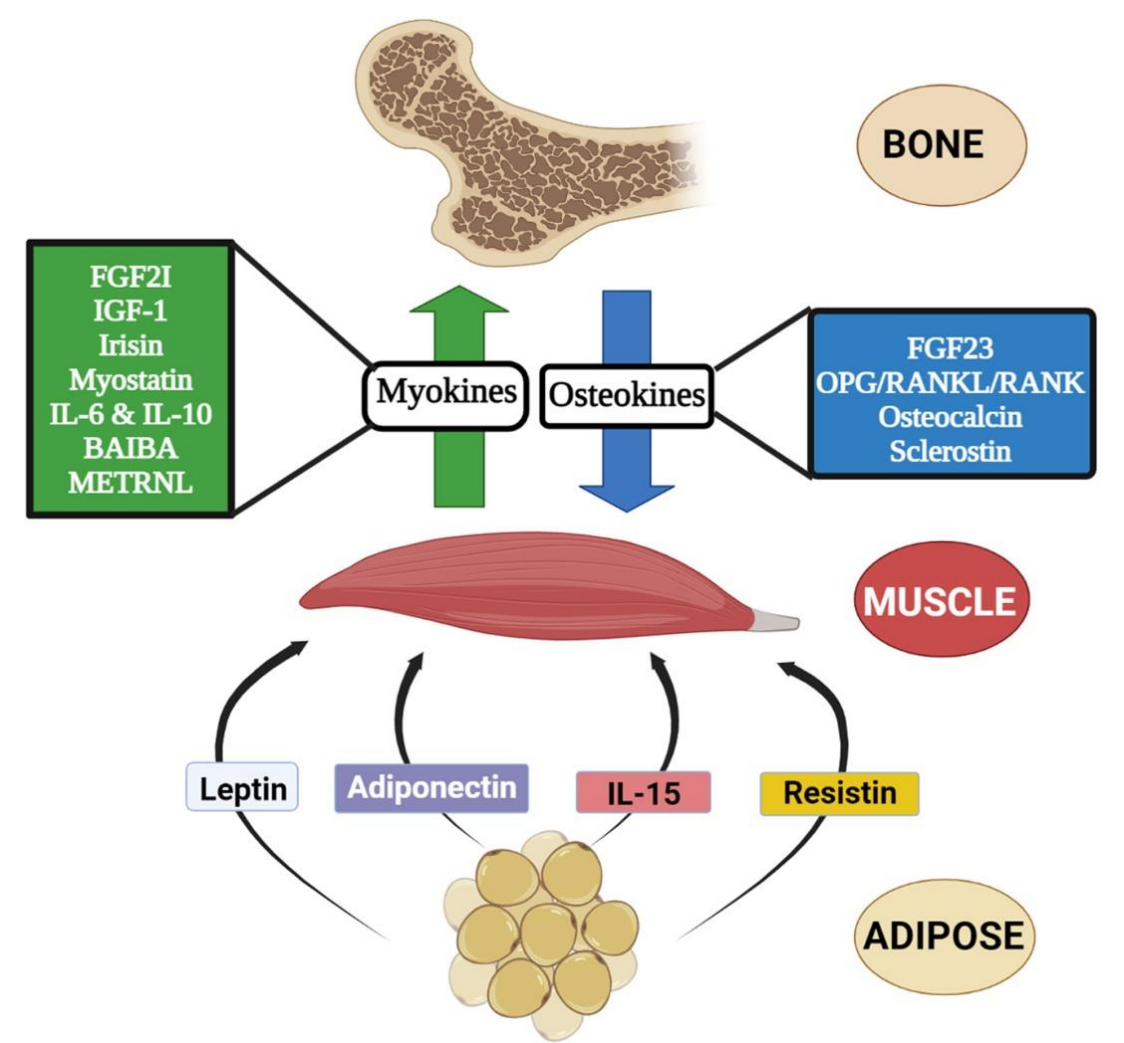
**The molecular crosstalk between bone, muscle, and adipose tissue by releasing myokines, osteokines, and adipokines**. The myokines like FGF21, myostatin, BAIBA, IL-6, and IL-10 are released from muscles affecting bone, whereas the osteokines like FGF23, osteocalcin, and sclerostin released from bones affect the muscles. Some adipokines like leptin, adiponectin, IL-15, and resistin released from the adipose tissues affect the muscles.

Muscles and bones are complementary to each other, and both of these components are exceptionally essential for individual autonomy and locomotion. The bones act like levers, which essentially help the muscle apply the force required for locomotion [73]. The two most common diseases observed in aging adults include sarcopenia and osteoporosis. However, later advancements in the research show that these two diseases are interconnected as muscles and bones are connected likewise. Thus, “osteo-sarcopenia” is a new term dealing with both diseases, characterized by a decrease in the density of bones and atrophy of the muscles. Recent advancements have vividly elaborated the interplay between the bone and muscles. However, the reciprocal relations of these two components should be addressed in detail to get a clear idea about the landscape of the various age-related musculoskeletal diseases [74]. Some of the myokines and osteokines that have a pivotal function in muscle-bone crosstalk in old-aged adults are discussed below (Fig. 4).

### 4.1. Myokines

Skeletal muscle has been widely recognized as a secretory organ in recent years. The cellular components of the muscles release certain chemical components, namely proteins and peptides. These factors are known as myokines. Myokines perform various biological tasks after being secreted by muscles, including regulating intricate endocrine, paracrine, and autocrine signaling pathways [75]. Sarcopenia, Type 2 diabetes, and obesity are some metabolic diseases associated with age [76-78]. The rate of secretion of the myokines decreases with aging, followed by an increase in the level of myostatin [79]. Some of the myokines involved in the crosstalk between bone and muscles in age-related disorders are discussed below.

#### 4.1.1. Fibroblast growth factor 21 (FGF21)

Fibroblast growth factor 21 (FGF21) is a pivotal factor in regulating muscle homeostasis [80]. The level of FGF21 increases as the person ages and exhibits some negative effects on bone density [81, 82]. The mechanism underlying this is the decision of bone marrow mesenchymal stem cells (BMSCs) to differentiate into adipocytes over the osteoclast or osteoblast cells. This bone resorption phenomenon occurs along the FGF21-IGFBP1 axis [83, 84]. Jung et al. reported that people suffering from sarcopenia have an elevated level of FGF21 in their serum, which in turn lowers muscle mass, resulting in poor gripping strength [85]. Studies have also proved that FGF21 is essential for the process of circulation in skeletal muscles. An excessive level of FGF21 in the blood serum causes premature aging followed by death. FGF21 increased levels in serum also cause systemic inflammation [86]. The elevated amount of FGF21 in serum can also help diagnose sarcopenia and thus can serve as a novel biomarker that has detrimental effects on the muscle cells [80].

#### 4.1.2. Insulin-like growth factor-1 (IGF-1)

The insulin-like growth factor-1 (IGF-1) has been found to be involved in many anabolic pathways regulating the skeletal muscles. Many isoforms of this IGF-1 molecule are associated with different functions in the skeletal muscles [87]. IGF-1 is important in the maintenance of skeletal muscle hypertrophy and strength. Furthermore, IGF-1 also inhibits the inflammatory response that might be encountered due to the toxin damage and increases the proliferation capacity of the satellite cells [88]. Besides, IGF-1 also reduces the mass of the skeletal muscles [89]. Three IGF-1 isoforms, namely IGF-1Ea, IGF-1Eb, and IGF-1Ec (also called MGF), can be produced by cleaving the IGF-1 gene. The damage in the skeletal muscle releases an IGF-1 isoform named MGF, which produces mucin and some calcium-dependent cell adhesion molecules. The MGF isoform helps in the activation of the satellite muscle cells. Consequently, throughout protein synthesis, IGF-1Ea expression is quite high. As a result, when muscles are injured, MGF is released, followed by the activation of the satellite cells, and the total amount of satellite cell reserve influences the ability of muscles to regenerate [90, 91]. Studies have provided evidence supporting the association between sarcopenia and reduced IGF-1 signaling, namely the isoform IGF-1Ec, also known as MGF. The MGF expression in skeletal muscle cells has been reported to be lower in individuals with sarcopenia [92, 93].

#### 4.1.3. Irisin

As a person ages, the effect on mobility with weakened muscle mass has been linked to decreased irisin levels. This finding is attributed to the relationship of irisin level with age-related disorders [94]. Irisin is a very important component in preserving the size and strength of skeletal muscles [95]. When the IGF-1/mTOR/Akt signaling pathway is downregulated by a compound named myostatin [96, 97], irisin helps activate the ERK and Akt signaling cascades by stimulating the IGF-1/mTOR/Akt pathway. Furthermore, introducing irisin in mice models also explicated irisin's capacity to induce muscle hypertrophy by the IGF-1/mTOR/Akt pathway, which simultaneously activates the synthesis of the muscle proteins [98]. The activation of ERK by irisin leads to the upregulation in the level of IGF-1, which decreases the level of myostatin in the muscle cells, highlighting irisin as a positive regulator for controlling the activities of the skeletal muscles [99]. Studies have also elucidated that postmenopausal woman diagnosed with sarcopenia had lower irisin concentrations than pre-sarcopenic subjects [100].

#### 4.1.4. Myostatin

One of the members of the TGF-β superfamily of proteins is myostatin which induces a negative control on muscle hypertrophy and is primarily expressed in the skeletal muscle [96, 99]. By activating the Smad molecules, which are considered to disrupt the Akt, IGF-1, and mTOR pathways, myostatin reduces the synthesis of proteins in skeletal muscles [96, 97]. Myostatin prevents protein synthesis, enhances FOXO-mediated muscle atrophy, and lowers muscle glucose uptake by blocking AMPK and GLUT4. An upregulation of myostatin may partially explain age-related muscular atrophy and diminished strength with advancing age [101]. Myostatin communicates with cells via the ActRIIB receptor leading to the formation of a heterodimer involving ALK5 or ALK4. These molecules contain some intracellular threonine or serine domains which phosphorylate the Smad molecules and form the Smad complex. The Smad complex moves inside the nucleus, mediates the cellular transcription of the genes required for cellular differentiation and proliferation, and degrades the muscular proteins in mature myofibers [102-104].

#### 4.1.5. IL-6 and IL-10

The probable involvement of various inflammatory cytokines in sarcopenia has drawn more attention in recent years. There is proof that sarcopenia is directly linked with age-related inflammation. Reduced muscle strength and muscle mass have been linked to a surge in the levels of IL-6 and IL-10 [105]. It has been proposed that some inflammatory cytokines, such as IL-6 and IL-10, cause muscle atrophy; High levels of IL-6 are associated with reduced muscle mass and muscle strength, and sarcopenia and sarcopenic obesity have drastically increased C-reactive protein levels [106]. Ferrucci et al.'s study elucidated the fact responsible for the comparable reduction in muscle strength may help to explain why older women with high IL-6 serum levels are more likely to exhibit physical disabilities and experience a steeper decline in their ability to walk than older women with lower levels [107]. According to recent research, inflammatory responses may potentially cause sarcopenia by inducing anomalies in the mitochondria that affect the origin or number of mitochondria [108].

#### 4.1.6. β-aminoisobutyric acid

β-aminoisobutyric acid (AIBA), like all other amino acids except glycine, comprises two enantiomers, namely L-BAIBA and D-BAIBA. The presence of BAIBA has been identified in urine, where it is produced as a myokine in response to physical activity. Additionally, BAIBA has been observed in mice with an overexpression of muscle peroxisome-proliferator-activated receptor-coactivator-1 (PGC1). The catalyst in question is a branched-chain amino acid that consists of thymine and valine. Its function involves the regulation of adipose tissue browning and enhancing fatty acid β-oxidation inside the liver. [109]. Treatment with BAIBA could prevent the death of osteocyte cells caused by reactive oxygen species. In addition to reducing adipose tissue mass and lowering the rate of inflammation in adipose tissue and skeletal muscles, BAIBA is engaged in the glucose homeostasis involved in regulating the subcutaneous white adipose tissue [109-114]. Younger persons have greater plasma BAIBA levels than older adults [115, 116]. It was discovered that the protective impact of BAIBA diminished with aging, most likely due to a decrease in the expression of the Mas-Related G Protein-Coupled Receptor Type D (implicated in portal hypertension-related splanchnic vasodilation in both cirrhotic and noncirrhotic portal hypertension) rather than a reduction in the muscle's ability to make BAIBA [117, 118].

#### 4.1.7. Meteorin-like Factor (METRNL)

A tiny protein molecule known as meteorin-like is thought to control inflammation, thermogenesis, and glucose metabolism [119]. It can downregulate insulin resistance and cause browning of the white adipose tissue. METRNL acts as an effective metabolic or immunological regulator of adipose tissue. Exercise and cold temperatures boost plasma levels of the hormone by enhancing its expression levels in adipose tissue and skeletal muscles. Increased levels of METRNL stimulate the browning of adipocytes. It's interesting to note that METRNL seems to work through immune system cells that invade adipose tissue rather than directly on adipocytes. It was discovered that METRNL works directly on macrophages through a Stat3-dependent mechanism. IGF-1 is subsequently activated, and an anti-inflammatory reaction is triggered, stimulating myogenesis along with the muscle satellite cells [120, 121]. METRNL is, therefore, said to have an impact on muscle regeneration and myogenesis. METRNL might also mediate to stimulate the interaction between muscles and bone during aging [74].

### 4.2. Osteokines

Recently, it has been discovered that a few substances produced by bone affect various tissues, including muscle, systemically. These factors are known as osteokines. Recently, more about the interaction between bone and muscles, apart from their principle of mechanical coupling, has been investigated. Both muscle and bone are regarded as secretory endocrine organs, and their interactions may alter how each of these organ’s functions. The osteokines and other muscle-derived substances can influence the metabolic activities of both bones and muscles through the endocrine, paracrine, and autocrine signaling pathways [122]. Some of the osteokines mediating the biochemical network between bones and muscles are summarized as follows:

#### 4.2.1. Sclerostin

The sclerostin molecule, derived from the osteocytes, is considered a putative myokine involved in sarcopenia [123]. Recent advancements in science delineated the involvement of sclerostin molecules in the interplay between the muscles and bone by inducing the Wnt signaling pathway capable of inhibitory effect [124]. Ota et al. cited that the sclerostin level was higher in old-aged mice models than the young ones, highlighting that increased sclerostin is inversely proportional to osteogenic activity, resulting in decreased bone formation [125]. Sclerostin has the ability to inhibit the cross-talk between muscle cells (C2C12) and MLO-Y4 osteocytes mediated by WNT3a, by regulating the Wnt/-catenin pathway. [126]. A population study conducted in Korea involving aged adults elucidated that the elevated level of sclerostin in the samples of patients suggests a lower chance to be affected by sarcopenia and lower muscle mass [127]. Besides, Ahn et al.’s hypothesis also put forward the fact that the level of sclerostin molecule decreases to a greater extent in sarcopenic patients suggesting it to act as a therapeutic agent or a potential marker for treating sarcopenia [127].

#### 4.2.2. Osteocalcin

A huge controversy exists in the scientific community regarding osteocalcin and aging. According to the theories of Mera et al. and Diemar et al., osteocalcin levels drop as a person age [128, 129]. It is the most widely present non-collagen protein distributed in the bone matrix and is secreted from the mature osteoblasts [130]. The osteocalcin present in the muscle is important for catabolism during exercise. At first, the glycogen is broken down through the osteocalcin signaling pathway in the muscle fibers. Moreover, it also enhances the uptake of GLUT4, which facilitates the process of glycolysis. The osteocalcin signaling pathway even helps to degrade and absorb fatty acid molecules [128]. Osteocalcin’s effect could be obscured by numerous subtypes, which account for conflicting outcomes in the downregulation of osteocalcin levels with age, followed by bone loss. This limits the use of osteocalcin for treating sarcopenia without a great advancement in this research [74]. The osteocalcin signaling pathway plays a crucial function in combating the severities in skeletal muscle tissues. However, it is imperative to investigate the potential interaction between osteocalcin and various molecules involved in muscle activity in order to ascertain its role in combating illnesses [131].

#### 4.2.3. OPG/RANKL/RANK

A transmembrane protein called RANKL is predominantly found in osteoblasts and osteocytes. To examine the association of RANKL with the aging process, it is crucial to correlate the OPG/RANKL/RANK system simultaneously [132, 133]. As individuals age, there is a decrease in the bone-protective factor known as osteoprotegerin (OPG). Conversely, researchers have observed a rise in the levels of receptor activator of nuclear factor kappa-B ligand (RANKL), receptor activator of nuclear factor kappa-B (RANK), and the ratio of RANKL to OPG within the bone marrow [134, 135]. Regarding muscle, RANKL/OPG/RANK has been linked to reports of muscular atrophy, but it has been observed far less frequently for sarcopenia. According to Bonnet et al., osteo-sarcopenic Pparb/mice and postmenopausal humans with impaired muscular function could benefit from the RANKL inhibitor Dmab [136]. This suggests that simultaneously treating osteoporosis and sarcopenia via the OPG/RANKL/RANK axis may be feasible and beneficial.

#### 4.2.4. Fibroblast growth factor 23 (FGF-23)

The first hormone-like osteokine released by the osteocytes is FGF-23 [137]. Similar phenotypes associated with premature aging, including heart hypertrophy [138, 139], cognitive impairment [140], arterial calcification [141], and metabolic bone diseases, were observed in FGF-23 knockout animal models. The elevated levels of FGF-23 in adults were reported according to a cross-sectional research study that involved 2977 aged adults. This finding indicates that FGF-23 might have unfavorable biological effects [142]. Furthermore, aside from facilitating the differentiation of co-cultured satellite cells into cells resembling muscle tissue [143], skeletal muscle mesenchymal stem cells (MSCs) also possess the ability to sustain the muscle fibers [144]. As a result, skeletal muscle homeostasis and regeneration may be significantly influenced by the interaction between MSCs from skeletal muscle and satellite cells [145].

The various myokines and osteokines having a critical role in sarcopenia are summarized in Table 1.

**Table 1:** The functions of different myokines, osteokines and adipokines are involved in the functioning of the muscle cells.

Mediator molecules	Factors	Function	Ref.
**Myokines**	Fibroblast growth factor 21 (FGF2I)	Maintains muscle homeostasis	[80]
	Irisin	Maintains the strength and mass of the skeletal muscles	[95]
	Insulin-like growth factor-1 (IGF-1)	Maintains the strength and mass of the skeletal muscles	[88]
	β-aminoisobutyric acid	Prevents the death of osteocyte cells caused by reactive oxygen species	[117]
	Myostatin	Deficiency of this molecules prevents muscle atrophy	[74]
	Meteorin-like Factor (METRNL)	Controls inflammation, thermogenesis, and glucose metabolism	[119]
**Osteokines**	RANKL	Maintains the OPG/RANKL/RANK pathway in the process of aging	[132, 133]
	Osteocalcin	Important for catabolism during exercise	[130]
	Fibroblast growth factor 23 (FGF-23)	Regulates the metabolism of phosphate and Vitamin D	[185]
	Sclerostin	Helps in modulating the mass of the muscles	[186]
**Adipokines**	Leptin	Maintains the energy homeostasis between the muscles and fat	[151]
	Adiponectin	Maintains muscle metabolism	[156]
	IL-15	Observed during the development of 3T3-L1 adipocytes and C2C12 myoblasts	[167]
	Resistin	It prevents myogenic differentiation	[184]

## 5. Molecules involved in adipose tissue-muscle crosstalk sarcopenia

Adipose tissue and skeletal muscle failure are mutually regulated and closely linked to age-associated obesity and sarcopenia [146]. The earliest indication of a connection between muscle mass and gain in adipose tissue comes from observing changes in muscle number and quality in people with both excess and insufficient adipose tissue [147]. An excess amount of adipose tissue may be linked to muscle injury through the increased generation of free fatty acid by the hypertrophic adipocytes that can ectopically aggregate between the muscle fibers [146]. Lipids and their derivatives tend to accumulate within and among muscle cells, resulting in the impairment of mitochondrial function, disruption of fatty acid β-oxidation, formation of ROS, development of insulin resistance, lipotoxicity, and heightened release of proinflammatory cytokines. While muscle-secreted cytokines or myokines have the potential to exacerbate adipose tissue atrophy, persistent low-grade inflammation, and initiate a harmful loop of local hyperlipidemia, they can also contribute to systemic inflammation and insulin resistance, promoting the progression of sarcopenic obesity [146]. This results in lipotoxicity, significantly contributing to the pathogenesis of sarcopenia, sarcopenic obesity, and insulin resistance [148].

Adipokines are a class of cytokines generated from adipose tissues that operate like hormones and control metabolic responses through paracrine, endocrine, and autocrine signaling [149]. Adipokines also influence bone turnover, including bone mineral density (BMD), skeletal muscle catabolism in aging adults, and their role in developing metabolic illnesses like sarcopenia and osteoporosis [150]. Some of the adipokines responsible for maintaining the crosstalk between adipose tissue and muscles are discussed below (Fig. 4).

### 5.1. Leptin

Leptin is crucial in controlling the energy balance between muscle and fat [151]. This proinflammatory adipokine also controls energy balance through the hypothalamus and is strongly related to overall body obesity [152]. Through AMPK regulation, leptin is hypothesized to control skeletal muscle [153]. Leptin infusion increases the size of the muscle fibers in animal models by activating the insulin signaling pathway [154]. The blood leptin level is reported to have a positive correlation with the risk of dynapenia, whereas the serum leptin level negatively correlates with the risk of sarcopenia in both female and male aged people. More detailed studies are required to prove the molecular reasons driving its favorable connection with muscle mass and its negative regulation of muscle strength [151]. Tazawa et al. reported that increased levels of leptin elevate the production of IL-6 and cause ectopic inflammation in skeletal muscles in old-aged rats, and this inflammation directly contributes to sarcopenia. Previous studies reported that the deposition of the adipose tissues in the muscles of aged individuals causes ectopic inflammation, whose mechanism of action is yet to be deciphered [155].

### 5.2. Adiponectin

It has been demonstrated that adiponectin can influence muscle metabolism and inflammatory state [156]. Low circulating adiponectin level is generally associated with abdominal obesity [157, 158]. Adiponectin controls skeletal muscle functions through the AMPK-stimulated translocation of GLUT4 and fatty acid oxidation. Adiponectin may also help maintain or grow muscle fiber size as we age by encouraging myogenesis in the satellite cells and preventing proteolysis [159]. However, it is unknown how adiponectin relates to sarcopenia, which mostly develops in aging individuals. Sarcopenia-related older persons have lower serum adiponectin levels than the non-sarcopenic subjects [160]. Several significant epidemiological studies, however, have also found correlations between high blood adiponectin levels and reduced muscle density [161, 162], the low muscular mass, poor functionality, and a higher chance of sarcopenia [163]. Moreover, sarcopenia development is also significantly influenced by the expression of particular skeletal muscle adipokine receptors [164]. The long isoform of the leptin receptor mediates the effects of leptin on skeletal muscle. Similar to AdipoR1, this leptin receptor's expression is decreased in the skeletal muscle of obese people [165]. Leptin receptor and AdipoR1 expression levels in skeletal muscle are decreased in obesity, and this likely affects how sensitive the muscle is to adipokine concentrations in the blood. Thus, older adults and adults who are obese may have lower levels of expression of AdipoR1 in skeletal muscle, which could contribute to sarcopenia [166].

### 5.3. IL-15

The adipose tissue and skeletal muscle generate IL-15, a cytokine belonging to the four-helix bundle family. Although at a noticeably lower level, it is also expressed in different stages of 3T3-L1 adipocytes and during the development of C2C12 myoblasts [167]. Muscle fibers and differentiated myocytes may be stimulated by IL-15 to assemble more contractile proteins [168, 169]. IL-15 in healthy older adults is 1.5-2 folds higher than in younger adults [170]. However, the expression of the IL-15 receptor signaling component IL2RB was found to be 80% lower in the older muscle cells, indicating that the effects of IL-15 on intracellular pro-myogenicity may be diminished in the elderly age [171]. Because of poor translation caused by many initiation sites of the AUG codon in the 5′-untranslated region, the mRNA levels of IL-15 do not always correlate with IL-15 protein expression [172]. Additionally, only one of the two mature isoforms created by the alternative splicing precedes the secretory pathway [173]. There may be a transcriptional attempt to compensate for some of the secretory capacity or impaired translation that gives rise to the phenomenon called inflammation in order to reduce the catabolic effects of the circulating IL-15, which may explain the main reason behind the increased level of IL-15 in aging adults along with the reduction in the levels of circulating IL-15 in sarcopenia [174].

### 5.4. Resistin

Initially, resistin was identified as a white adipose tissue-produced adipokine that causes insulin resistance in rats [175]. It is a proinflammatory cytokine that is mostly produced by inflammatory cells like macrophages, neutrophils, and monocytes in humans [176]. By turning on the traditional NF-κB pathway, resistin can potentially interfere with myotube function and nuclear fusion [177]. Resistin also prevents myogenic myoblast development [178]. Insulin signaling [179], a key regulator of muscle development and regeneration [180], has been demonstrated to be impaired by resistin. There is a dearth of in vivo studies on how resistin regulates skeletal muscle hypertrophy and function [181]. The plasma resistin levels, on the other hand, have been found to correlate with aging [182]. Resistin levels were discovered to be adversely linked with quadriceps strength and muscle size [183]. A decrease in skeletal muscle mass results from resistin's promotion of adipocyte differentiation and inhibition of the satellite cells to differentiate into skeletal muscle cells [184].

The various adipokines released from the adipose tissue and having a role in sarcopenia are summarized in Table 1.

## 6. Molecular crosstalk in aging neuromuscular junctions during sarcopenia

Muscle wasting is a quick and progressive disorder. Muscle strength is significantly lost more than two-fold compared to muscle size [187]. Indeed, the anatomy of the neuromuscular junction (NMJ) changes an individual's age, which might disrupt the interplay between the neurological and muscular systems. The rate at which denervated fibers form exceeds the pace of reinnervation in elderly persons. This may be due to a decline in the ability to improve reinnervation-related processes, such as the neural cell adhesion molecule (NCAM) response. The preservation and functioning of the NMJ depend on many signaling pathways involving diverse components. The aforementioned components are of utmost importance in upholding the stability of both the pre- and postsynaptic apparatus, as well as facilitating the accurate transmission of signals mediated by Acetylcholine (Ach). These signaling pathways are also involved in reinnervation that occurs after denervation. However, the extent to which NMJ dysfunction contributes to sarcopenia development is still not fully understood [188]. This is an important subject that warrants more investigation to enhance the therapy of sarcopenia. A significant reduction in specific force can occur when the use of individual skeletal muscle fibers is reduced [189, 190]. It is difficult to fully understand the significant difference (more than two-fold) between muscle force loss and muscle mass for these reasons alone such as reduction in the number of actomyosin cross-bridges, calcium (Ca^2+^) alterations [191], decreased concentration of myosin and change of myosin function by post-translational modifications in muscle fibers [192]. Control change in NMJ and somatomotor might be involved in the cause of unbalanced force loss[193]. Certainly, maladaptations by inactivity can be found in the motor system like a motoneuron, cortex, NMJ function, and corticospinal excitability [194], and could induce the alteration of motor unit discharge [195].

The NMJ is one of the peripheral synaptic connections between the skeletal muscle and the terminals of the motor nerve. It plays an important role in controlling muscle contraction because it is the site for transmitting action potential from the nerve to the muscle [193, 196]. Furthermore, the interaction of muscle fibers and terminals of motor nerves is crucial for the formation of NMJ. One of the factors that mediate the process of peripheral synapsis is Agrin. It is utilized by motor neurons [197]. It triggers the activity of the transmembrane tyrosine kinase that is specifically present in skeletal muscles. The mutant mice that lack either skeletal muscles or agrin do not develop NMJ, suggesting the vital importance of both [198, 199]. The maintenance of NMJ is essential to regulate muscle mass; thus, there is a growing curiosity in investigating the molecular processes responsible for the reduction of muscle mass due to the absence of nerve stimulation. mTOR signaling, in other words the activation of mTORC1 in the skeletal muscle fibers is tremendously important for maintaining the fiber innervation in a proper orientation and preserves the structural integrity of the NMJ in the motor neuron as well as muscle fiber [200]. Indeed, the absence of both Raptor as well as mTOR, or the application of rapamycin, can effectively trigger the emergence of (NCAM)-positive fibers, which indicates the occurrence of fiber denervation and deterioration of neuromuscular junctions. Significantly, extended mTORC1 deprivation results in muscle fibrillation and notable fragmentation of the NMJ. However, the employment of a peptide molecule named Tat-beclin1 can help in reactivating autophagy and impede mitochondrial dysfunctioning followed by the resemblance of NCAM-positive fibers. Taken together, these findings demonstrate that the essential upkeep of the neuromuscular junction (NMJ) relies on the foundational mTORC1 signaling within fully developed muscle fibers.

Skeletal muscle fiber-derived extracellular signal-regulated kinases 1 and 2 (ERK1/2), which are common and fundamental intracellular mitogen-activated protein kinases, play a regulatory function in upholding the neuromuscular junction and the establishment and safeguarding of skeletal muscle fiber characteristics [201]. From the brain to the neuromuscular connections, sarcopenia is related with a variety of neurological problems that affect our capacity to carry out deliberate movements. A decrease in signals from the upper brain centres is caused by decreased dopamine activity, inadequate motor planning, and poor coordination. Slower conduction rates and lessened muscular responsiveness are caused by modifications to the organisation of motor units and inflammatory processes that impact motor neurons. The reorganisation of neuromuscular connections and alterations in neurophysiology brought on by ageing may potentially contribute to problems with the neuromuscular system [188]. WNTs have been shown to regulate AGRIN-induced AChR clustering in vitro [202, 203]. Moreover, LRP4 have interation between agrin and skeletal muscle [204, 205]. It is also evident that both homozygous LRP4mte and LRP4mitt experience NMJ (neuromuscular junction) issues akin to those found in skeletal muscle mutant mice. These issues encompass irregular growth and division of motor axons, the absence of AChR clusters formed without neural influence as well as nerve-induced clusters, and the nonexistence of gene expression specific to synapses [206] Several potential biomarkers, including the brain-derived neurotrophic factor (BDNF) and C-terminal agrin fragment (CAF), as well as some therapeutic targets such as calcitonin gene-related peptide (CGRP) and ACh, have been identified. It might pave the way for future research to validate their clinical utility.

## 7. Advances in therapeutic interventions for sarcopenia

With the rising aging of the global population, sarcopenia will emerge as a major clinical issue affecting millions of elderly people. According to the Food and Drug Administration (FDA), it is hard to differentiate whether patients have natural aging or sarcopenia [207]. As a result, elderly people should understand that sarcopenia is a disease that can be prevented or treated. Therefore, it is essential for healthy aging that we develop numerous therapeutics and preventive approaches against sarcopenia. Since the clinical symptoms and causes differ between patients, future large-scale clinical studies are crucial for developing precision medicine that considers the different symptoms of each person [208]. However, sarcopenia trial design is difficult yet beneficial since elderly peoples’ progressive, debilitating process can be stopped. The lack of a widely accepted terminology and consistent evaluation methodology hinders current research on the likely molecular pathways responsible for initiating sarcopenia [209]. Despite tremendous advances in understanding the multiple origins of sarcopenia, the majority of therapies have focused on ameliorating the environmental causes of sarcopenia, namely increasing physical activity and providing proper nutrition. Clinical intervention studies have demonstrated that nutrition and resistance training can considerably enhance functional results in even the most aged and frail nursing home residents [210]. In addition, recent clinical trials have shown considerable increases in muscle protein synthesis in older individuals who engage in physical exercise and a healthy diet [211]. A few clinical trials that have been conducted for the treatment of sarcopenia are listed in Table 2.

**Table 2:** Clinical phase trials that have been completed or are undergoing for treating Sarcopenia.

DRUGS	TRIAL NO.	PHASE	STATUS
**Cetylpyridinium Chloride (CPC)**	NCT02575235	Early Phase 1	Completed
**Bimagrumab**	NCT02333331	Phase 2	Completed
**L-NMMA**	NCT00690534	Phase 1	Completed
**MK-0773**	NCT00529659	Phase 2	Completed
**Omega-3 fatty acids**	NCT02103842	Phase 1	Completed
**REGN1033**	NCT01963598	Phase 2	Completed
**BPM31510**	NCT04999488	Early Phase 1	Not yet recruiting
**Allopurinol**	NCT01550107	Phase 4	Completed
**MYMD-1**	NCT05283486	Phase 2	Recruiting
**Losartan**	NCT01989793	Phase 2	Completed
**Tadalafil**	NCT05458232	-	Recruiting
**Creatine Monohydrate**	NCT03530202	Phase 2	Unknown
**Rapamycin**	NCT00891696	Phase 1	Completed
**GLP-1**	NCT02370745	-	Completed
**BIO101**	NCT03452488	Phase 2	Completed
**Anamorelin Hydrochloride**	NCT04021706	Phase 1	Active, not recruiting
**Pioglitazone**	NCT02305069	-	Completed
**Rapamune**	NCT05414292	-	Recruiting

Recently, the panorama of intellectual property (IP) in the field of innovation has been expanding daily. Pharmaceutical corporations require patent protection, and pharmaceutical businesses invest millions of dollars in their research [212]. Like many other diseases, many patents are filed to prevent sarcopenia; some are listed in Table 3.

**Table 3:** The patents related to sarcopenia.

Patent no.	Application no.	Content of the patent	Inventors	Applicant
Patent type	Publication date			
**US8835485B2**	US13/263	The prevention of sarcopenia by administration of a substance that both reduces the sensibility of *beta-adrenergic receptors and of 5-HT1a receptors*	Jochen Springer, Stefan Anker, Andrew Coats, John Beadle	Myotec Therapeutics Ltd., Actimed Therapeutics Ltd.
**United States**	September 16, 2014			
**US9301941B2**	US13/499	The invention prevents sarcopenia and muscle atrophy in animals by administering *isoflavones* to the animals.	Yuanlong Pan, Sunil Kochhar, Serge Andre Dominique Rezzi, Francois-Pierre Martin, Emma Peré-Trepat, Sebastiano Collino, Francia Arce Vera	Societe des Produits Nestle SA
**United States**	April 5, 2016			
**WO2016092439A1**	PCT/IB2015/059369	Administration of *Myostatin or activin antagonists* for the treatment of sarcopenia	Patrick KORTEBEIN, Daniel ROOKS, Lloyd B. Klickstein, Ronenn Roubenoff, David Glass, Estelle Trifilieff, Dimitris PAPANICOLAOU	-
**WIPO (PCT)**	June 16, 2016			
**WO2015151066A1**	PCT/IB2015/052453	Treatment of sarcopenia with *ecdysteroid*s	Miguel Jimenez Del Rio, Jose Maria ZUBELDIA FERNANDEZ, Aaron HERNANDEZ SANTANA, Julia Charlotte WIEBE	-
**WIPO (PCT)**	October 8, 2015			
**WO2006083183A1**	PCT/NZ2006/000010	Use of *myostatin (gdf-8) antagonists* for the treatment of sarcopenia	Ravi Kambadur, Mridula Sharma, Alex Hennebry, Monica Senna Salerno de Moura	-
**WIPO (PCT)**	August 10, 2006			
**US7442706B2**	US7442706B2	Methods for treating sarcopenia with a *growth hormone secretagogue*	Michael O. Thorner	Lumos Pharma Inc.
**United States**	October 28, 2008			

## 8. Targeting muscle-bone-adipose crosstalk for therapeutic intervention

Until now, therapeutic approaches for preventing fractures associated with sarcopenia have only focused on the bone; however, a growing understanding of muscle-bone-adipose tissue crosstalk may represent a new treatment paradigm for sarcopenia. It infers that cutting-edge therapeutical strategies should focus on factors involved in crosstalk between bone, muscle, and adipose cells. The activin signaling system, known for its significant role in inhibiting bone and muscle development, has emerged as a promising therapeutic target for sarco-osteoporosis. This is evident from studies demonstrating that myostatin inhibitors effectively enhance muscle and bone mass in young adult mice [213, 214]. The administration of ActRIIB-Fc, a soluble receptor for myostatin, to mice has been observed to enhance bone synthesis alongside the anticipated advantages for muscle. This finding implies that this approach has a direct impact on the activity of osteoblasts. The present study employed a double-blind, placebo-controlled design to investigate the effects of a single dosage of ActRIIB decoy receptor on lean mass in a sample of 48 postmenopausal women. Results revealed a substantial increase in lean mass following the administration of the intervention after one month. Furthermore, the administration of ActRIIB therapy resulted in an elevation of bone turnover serum biomarkers, such as C-terminal telopeptides (CTX) and ALP [215]. Using such anabolic agents can potentially avert age-related sarcopenia and osteoporosis by providing dual benefits that positively affect both muscle and bone. Further clinical studies are required to determine other such inhibitors in the activin pathway that can provide long-term safety and effectiveness. In the near future, osteocalcin and irisin could be possible targets for treating sarcopenia and osteoporosis, as they are two of the many factors engaged in the crosstalk between muscle and bone [214].

Vitamin D is a hormone that exerts its effect on both muscular and skeletal systems. It has been observed to have wide-ranging impacts, such as its influence on calcium homeostasis, as well as its role in the differentiation and growth of muscle and bone cells. Moreover, Vitamin D regulates hormones released from muscle and bone, potentially enabling communication between these bodily tissues. In the clinical setting, it has been discovered that a deficiency in vitamin D or mutations affecting the vitamin D receptor can result in a comprehensive deterioration of both muscle and bone tissues. This suggests a synchronized influence of vitamin D on these specific anatomical locations [216, 217]. As the body ages, the muscle and bone unit experience a reduction in the expression of Vitamin D receptors (VDR) at a local level. This is accompanied by the activation of proteolytic pathways within the muscle, leading to increased bone resorption and infiltration of adipose tissue. Both effects are influenced by the shared effects on PPARγ2 [218, 219]. For a long time, there has been a question about whether vitamin D's impact on bone and muscle is solely due to regulating calcium and phosphate levels or if it is partially influenced by the presence of local vitamin D receptors (VDR). The presence of VDR in muscle and bone is still debated, and transgenic mouse models haven't provided a conclusive answer. However, the effectiveness of vitamin D analog, eldecalcitol, has been demonstrated in elderly patients through concurrent improvements in both muscle and bone indices, as well as functional advantages [220]. Therefore, additional investigation is necessary to clarify the fundamental processes implicated in the tissue-modulatory and pleiotropic effects of vitamin D. Furthermore, it is imperative to investigate the therapeutic potential of targeting this route for treating musculoskeletal ailments.

Studies indicate that the onset of sarcopenia, characterized by the weakening of muscles and loss of mass, is linked to heightened immune cell infiltration and inflammation in adipose tissue. This inflammation has the potential to trigger a more extensive systemic inflammation [221]. Obesity induces a significant inflammatory reaction in adipose tissue, which has a self-sustaining nature and generates a detrimental cycle characterized by local inflammation mediated by paracrine/autocrine signaling and systemic inflammation regulated by endocrine mechanisms. This inflammatory response specifically targets skeletal muscle. Regarding the impact of obesity on sarcopenia, there exists a proposition that an asymmetry between inhibitory factors involved in muscle growth, such as TGFβ, myostatin, activins, and others, in comparison to stimulatory factors like BMPs, BDNF, irisin, and FST, plays a substantial role in the development of sarcopenia [222]. Findings indicate that these compounds play a significant role in regulating inflammation in skeletal muscle and adipose tissue. For instance, Dong et al. observed that myostatin and activin A, which are known to impede muscle growth, exhibit elevated levels in the aging process [223]. These molecules have been found to facilitate certain crucial processes linked to obesity while promoting the development of adipose tissue and inflammation in adipose tissue and skeletal muscle. On the contrary, it has been noted that muscle growth stimulants such as BMPs, BDNF, irisin, and FST, which are synthesized by adipose tissue and/or skeletal muscle, have anti-inflammatory characteristics [224, 225]. This finding confirms the notion that a robust correlation exists between the metabolism of skeletal muscles, adipose tissue, and inflammation.

There may potentially exist alternative mechanisms. An interesting study suggests that adipose stem cells derived from obese individuals have the ability to create a pro-inflammatory environment (activation of Th17 cells and monocytes) [226]. Consequently, this inflammatory environment inhibits adipogenesis and impairs adipocytes' insulin response. Furthermore, it has been suggested that the dysfunction of muscle stem cells (satellite cells), which occurs with age, may play a role in the muscular pathology associated with excess adipose tissue[227]. It can be observed that these impairments in satellite cells could significantly contribute to the development of sarcopenic obesity. Recent research suggests there may be a connection between chronic low-grade inflammation and the development of frailty as people age and experience sarcopenia. It's crucial to understand the molecular mechanisms that contribute to age-related obesity and sarcopenia so we can differentiate the causes and consequences of these conditions and improve strategies for preventing and managing them.

Skeletal muscle, a key endocrine organ, releases extracellular vesicles to regulate homeostasis and metabolism in addition to myokines. A considerable quantity of extracellular vesicles (EVs) circulate in the bloodstream, and among them, muscle-derived EVs account for around 1-5% of the overall circulating EV population. During physical activity, EVs that carry microRNAs (miRNAs) specific to muscle tissue are released into the bloodstream. It has been observed that approximately 60-65% of these muscle-specific EVs are positive for CD81 [228]. EVs play a crucial function in intercellular communication [229]. Few investigations have found that muscle EVs affect bone. Previous studies have demonstrated that EVs derived from C2C12 myoblasts or myotubes have the ability to enhance the osteogenic differentiation of MC3T3-E1 cells. This effect is achieved through activating the Wnt signaling pathway, wherein miR-27a-3p plays a crucial role as a functional component [230]. In addition, myoblast development was promoted by miR-27a [231], and a miR-27a mimic could prevent muscle atrophy caused by chronic renal disease [232], suggesting that miR-27a in muscle-derived EVs may play a role in the crosstalk between bone and muscle. Aside from the process of bone production, muscle EVs also have a detrimental impact on the formation of osteoclasts and mitochondrial metabolism, which is essential for developing mature osteoclasts. Most seniors exercise less frequently and less intensely, and EVs made from senescent cells have a different payload, leading to an age-related loss in somatic function. It is suggested to explore the potential therapeutic significance of muscle-specific miRNAs inside muscular EVs that undergo changes in response to exercise, specifically miR-1 and miR-133a, in managing osteo-sarcopenia.

Similarly, several miRNAs that appear to be changed in EVs from senescent bone cells have been identified to affect bone and muscle metabolism [74]. In young and aged BMSCs, a subset of differently expressed EV-miRNAs was investigated [233-235]. Some of the miRNAs that are downregulated are miR-24, miR-328, miR-365, and miR-374. It is believed that decreased miRNAs in EVs derived from BMSCs during aging may play a role in muscle and bone development, or at least muscle growth. miRNAs that have been found to be elevated include miR-15b, miR-17, miR-20a, miR-186, miR-221, miR-31a-5p, and miR-99b. Although numerous research studies have demonstrated the impact of modified miRNAs in EVs on the development of muscles and bones, further investigations are necessary to ascertain the potential contribution of the molecules mentioned above to the interplay between bone and muscle during aging [74]. Apart from BMSCs, elderly osteoclasts were observed to release EVs and have higher expression of miR-214-3p than young osteoclasts. The upregulation of miR-214-3p facilitates the direct differentiation of osteoclasts. Additionally, miR-214-3p has been detected in EVs produced from osteoclasts and is associated with reduced bone production [236]. Furthermore, Davis et al. observed that miR-183-5p exhibits an age-dependent increase and is present in EVs formed from bone tissue. These EVs were obtained from interstitial fluid samples collected from the bone marrow of mice at two distinct stages of their lifespan. Endocytosis of EVs by BMSCs causes decreased osteogenic differentiation and induced BMSC senescence [233].

Adipose tissue is well recognized as a crucial element in inter-organ communication, particularly with regard to skeletal muscle, owing to its substantial secretory capabilities. EVs are useful for transferring various substances across cells, and these molecules can affect the recipient cell's physiological and pathological processes. The field has paid much attention to the adipose-secreted EVs' regulatory mechanisms and cellular interactions with skeletal muscle because of their sustaining functions. EVs generated from different adipose cell types (preadipocytes, adipocytes, adipose tissue stem cells (Adipose-derived mesenchymal stem cells, ADSCs, adipose tissue macrophages (ATM), etc.) possess diverse characteristics that affect skeletal muscle differently. Moreover, the investigation of adipose-derived EVs on skeletal muscle is mostly focused on the payloads (proteins and miRNAs) included in the EVs [237]. The present work conducted an investigation wherein the transplantation of perimuscular adipose tissue (PMAT) into the hindlimb thigh muscles of young mice resulted in a reduction in the integrin α7/CD29-double positive muscular stem/progenitor cells population. This effect was found to be mediated by EVs formed from PMAT. Additionally, it was noted that the Let-7d-3p miRNA, which specifically targets HMGA2, a crucial transcription factor involved in the self-renewal of stem cells, showed the ability to suppress cell proliferation in muscle stem/progenitor cells and impede the overall molecular processes occurring in aged adipocytes. The accumulation of Let-7d-3p in aged PMAT EVs is attributed to the diminished expression of Lin28 A/B and nuclear factor-kappa B signaling [238]. Metabolic abnormalities affecting adipose tissue typically lead to metabolic diseases in skeletal muscle. Recent evidence suggests that miRNAs can be transferred directly to skeletal muscles through EVs produced from adipose tissue, negatively affecting gene expression. For instance, miR27a is highly expressed in the sera of obese individuals with type 2 diabetes, which could be derived from EVs released by adipose tissue. miR-27a generated from 3T3-L1 adipocytes promotes insulin resistance in C2C12 cells by targeting PPAR, a powerful modulator of whole-body lipid metabolism and insulin sensitivity [239]. Likewise, *in vitro* investigations have shown that PPARγ is involved in ATMs-EVs miR-155 mediated inhibition of insulin-stimulated glucose uptake in L6 muscle cells [240].

The translation of EVs mRNAs into proteins within recipient cells suggests that EVs carrying mRNAs derived from adipocytes have the potential to be transferred to recipient cells, hence facilitating the recipient cells' protein synthesis processes. EVs containing mRNAs derived from adipocytes have the potential to be transferred to target cells. Once internalized, these EVs can contribute to the protein synthesis processes of the recipient cells. This is because the exosomal mRNAs within these EVs can undergo translation into proteins within the recipient cells [241]. It's interesting to note that EVs generated from adipocytes *in vitro* include mitochondrial DNA [242]. However, the functions of the aforementioned adipocyte-derived EVs RNAs on skeletal muscle are yet unknown, necessitating additional research to understand their roles in adipocyte-myocyte interaction better. EVs released by ADSCs with the capability for multiple differentiation have the potential to regenerate and protect skeletal muscle. The protein profiles of ADSC-EVs were investigated by Ni et al., revealing the involvement of around 1466 proteins in several cellular activities. EVs derived from ADSC-EVs have been found to possess several proteins that exhibit the capacity to induce skeletal muscle growth and facilitate regeneration processes. These proteins achieve these effects by activating signaling pathways closely involved with skeletal muscle development, including the PI3K-Akt, Jak-STAT, and Wnt pathways [237, 243]. These findings suggest that adipose tissue-derived mRNAs, DNA, miRNAs, and proteins in EVs may majorly contribute to sarcopenia, an age-related muscle-wasting condition. Table 4 summarizes EVs originating from bone, muscle, and adipose tissues, which are potentially involved in crosstalk and have significant implications in advancing therapeutic strategies for sarcopenia.

**Table 4:** Extracellular vesicle constituents associated with bone-muscle-adipose crosstalk in sarcopenia.

Sites of release	Extracellular vesicle constituents	Functions	Ref.
**Bone**	miR-24	Enhances bone formation and myogenesis	[244]
	miR-328	Enhances bone formation and myogenesis	[244]
	miR-365	Enhances bone formation and myogenesis	[244]
	miR-374	Enhances bone formation and myogenesis	[244]
	miR-27a-3p	Enhances the process of osteogenic differentiation by regulating the β-catenin signaling pathway	[245]
	miR-214	Acts as a positive regulator of myoblast differentiation	[246]
	miR-15b	Acts as a negative regulator of myoblast differentiation and represses the process of osteogenesis	[247, 248]
	miR-17	Acts as a negative regulator of myoblast	[247]
	miR-20a	Acts as a negative regulator of myoblast	[247]
	miR-186	Acts as a negative regulator of myoblast	[247]
	miR-221	Acts as a negative regulator of myoblast	[247]
	miR-31a-5p	Acts as a negative regulator of myoblast	[247]
	miR-99b	Acts as a negative regulator of myoblast	[247]
	miR-15b	Acts as a negative regulator of myoblast	[247]
	miR-221	Hinders the process of myotube formation and has a negative effect on osteogenesis	[249]
	miR-183-5p	Blocks the process of osteogenic differentiation and causes senescence in BMSCs	[233]
**Muscle**	ITGB1	Fusion of myoblasts	[250]
	CD9	Fusion of myoblasts	[250]
	CD81	Fusion of myoblasts	[250]
	NCAM	Fusion of myoblasts	[250]
	CD44	Fusion of myoblasts	[250]
	Myoferlin	Fusion of myoblasts	[250]
	miR-27a	Acts as a positive regulator of myoblast differentiation	[231, 251]
**Adipocyte**	FASN, G6PD, ACC	Effect of lipogenesis (de novo)	[252]
	Let-7d-3p	Prevent the growth of cells by targeting HMGA2	[238]
	Laminins, Reelin, PEDF	Stimulates the development of skeletal muscle	[243]
	Adiponectin, FABP4	Impacts on the signaling processes and metabolic actions involved in adipocyte-muscle crosstalk	[253]
	FASN, G6PD, ACC	Effect of lipogenesis (de novo)	[252]

## 9. Conclusions and future perspectives

Sarcopenia is a major consequence of aging and continues to be a significant clinical concern affecting millions of older people. Sarcopenia is a medical condition that results from an imbalance between protein synthesis and degradation, causing a gradual decline in muscle mass. This ultimately leads to reduced muscular functionality. Sarcopenia is a multifactorial aging effect that includes chronic inflammation, NMJ dysfunction, and degenerative illnesses. The presence of age-related co-morbidities and muscle impairments poses challenges in the precise diagnosis of sarcopenia and in differentiating it from other forms of atrophic disorders. To date, physical activity, particularly when combined with nutritional supplements, is regarded as the sole effective strategy for managing sarcopenia and preventing its negative effects. Besides, no pharmaceutical approaches provide conclusive proof of their capacity to prevent the deterioration of physical function, leading to sarcopenia. Future pharmacological and clinical trials and epidemiological studies may drastically alter our understanding and therapeutic approach to treating older mobility impairment. Advanced cell-based analysis and human/animal research will offer insight into developing novel therapeutic approaches by providing a deeper knowledge of sarcopenia's molecular and cellular pathways. The majority of intracellular signaling pathways involved in muscle homeostasis are altered and might thus be explored as intervention targets for preventing, delaying, or correcting sarcopenia.

From the beginning of embryogenesis, growth and development, and even aging, bone and muscle are inextricably connected with both the shape and function of the body. However, the concept that bone and muscle work as endocrine organs that secrete substances that influence each other's functioning poses a challenge to the prevailing belief that their interaction is solely mechanical. Moreover, with aging, adipose inflammation causes fat transfer to the intra-abdominal region (visceral fat) and fatty infiltrations in skeletal muscles, leading to a decline in overall strength and function.

The significance of adipose infiltration into muscle tissue in the context of aging and the development of sarcopenia is another factor that must be considered. The existing body of evidence has conclusively shown that this phenomenon has a detrimental impact on muscle function and increases an individual's vulnerability to mortality, including bone fractures or cardiometabolic disorders like type 2 diabetes. Nevertheless, the precise molecular mechanisms behind the impact of intramuscular fat depots on muscle cell metabolism in instances of muscle wasting and the signaling between fat cells and muscle fibers remain to be fully understood. In addition, intramuscular lipids and their derivatives have a detrimental effect on mitochondrial function. Moreover, the increased secretion of pro-inflammatory myokines may play a role in developing muscle dysfunction. This process may contribute to a self-perpetuating cycle that sustains inflammation in both adipose tissue and skeletal muscle. The lipotoxic effects on skeletal muscle can be attributed to the presence of fat-derived factors and mitochondrial damage. However, further investigation is required to clarify the underlying mechanisms.

Another major hurdle is to develop dual muscle bone therapy. The precise description of sarcopenia is still poorly understood, and it is necessary to establish functional objectives and markers related to the musculoskeletal unit to validate favorable outcomes in efficacy trials. Concerns have also been expressed regarding the safety of these medications, specifically the off-target effects associated with manipulating ubiquitous signaling pathways. The progress of inhibiting the activin pathway has been impeded due to the presence of adverse effects such as telangiectasia, hemorrhage, and gonadotropin suppression [215]. Recognizing the musculoskeletal system's integrated biology and the intricate signals involved in bone/muscle aging (occasionally with modern-day obese status) requires developed drugs to be more specific and devoid of any side adverse effect other than the sarcopenia-affected bone-muscle unit.

Based on sarcopenia's known molecular processes, several in vitro models have been proposed for its investigation. Nevertheless, sarcopenia's etiology encompasses intrinsic pathways and extrinsic factors associated with the skeletal muscle microenvironment. Hence, utilizing in vitro models is not without inherent limits despite the numerous advantages associated with these models. In particular, skeletal muscle cells derived from donors with diverse biological conditions, including aging and insulin resistance, are ideal in vitro models for studying sarcopenia and associated diseases. Furthermore, a suitable in vitro model would be desirable for screening molecular targets or discerning therapeutic molecules. Animal models that exhibit natural aging or have been genetically engineered offer several advantages compared to in vitro models when studying the systemic consequences of sarcopenia, molecular patterns at the organ level, and biochemical biomarkers. Nevertheless, the utilization of animals proves to be either inefficient or time-consuming. Thus, further research should be done toward developing state-of-the-art organ chips (Human-Derived Organ-on-a-Chip) or organoid models for sarcopenia, which may be more reliable and easier to screen the potent therapeutic molecules.

Thus, muscle-bone-adipose tissue crosstalk-based therapeutic approaches are a hotspot for investigation and systemic approach to signal modifying small compounds, medication repositioning (drug repurposing), and the recent discovery of muscle-specific extracellular vesicles (mRNA, peptides, miRNAs) are all part of the new strategies to understand and manage sarcopenia. In conclusion, the interest in sarcopenia must be increased, and the interventions for sarcopenia should be continually developed to understand molecular crosstalk among muscle-bone-adipose tissues, particularly by analyzing the role of secretome between these intricate tissues.
